# Development and pilot test of a peer-support based Cardiac-Diabetes Self-Management Program: A study protocol

**DOI:** 10.1186/1472-6963-11-74

**Published:** 2011-04-11

**Authors:** Chiung-Jung Jo Wu, Anne M Chang, Mary Courtney, Lillie M Shortridge-Baggett, Karam Kostner

**Affiliations:** 1 School of Nursing and Midwifery, Institute of Health and Biomedical Innovation, Queensland University of Technology, Victoria Park Road, Kelvin Grove, QLD 4059, Australia; 2 Mater Health Services, Brisbane, Australia; 3 Health and Social Development, University of British Columbia Okanagan, Kelowna BC V1V 1V7, Canada; 4 Department of Graduate Studies, Lienhard School of Nursing, Lienhard Hall 309 Pace University, 861 Bedford Road, Pleasantville, New York 10570-2799, USA; 5 School of Medicine, University of Queensland, Australia

## Abstract

**Background:**

People with cardiac disease and type 2 diabetes have higher hospital readmission rates (22%) compared to those without diabetes (6%). Self-management is an effective approach to achieve better health outcomes; however there is a lack of specifically designed programs for patients with these dual conditions. This project aims to extend the development and pilot test of a Cardiac-Diabetes Self-Management Program incorporating user-friendly technologies and the preparation of lay personnel to provide follow-up support.

**Methods/Design:**

A randomised controlled trial will be used to explore the feasibility and acceptability of the Cardiac-Diabetes Self-Management Program incorporating DVD case studies and trained peers to provide follow-up support by telephone and text-messaging. A total of 30 cardiac patients with type 2 diabetes will be randomised, either to the usual care group, or to the intervention group. Participants in the intervention group will received the Cardiac-Diabetes Self-Management Program in addition to their usual care. The intervention consists of three face-to-face sessions as well as telephone and text-messaging follow up. The face-to-face sessions will be provided by a trained Research Nurse, commencing in the Coronary Care Unit, and continuing after discharge by trained peers. Peers will follow up patients for up to one month after discharge using text messages and telephone support. Data collection will be conducted at baseline (Time 1) and at one month (Time 2). The primary outcomes include self-efficacy, self-care behaviour and knowledge, measured by well established reliable tools.

**Discussion:**

This paper presents the study protocol of a randomised controlled trial to pilot evaluates a Cardiac-Diabetes Self-Management program, and the feasibility of incorporating peers in the follow-ups. Results of this study will provide directions for using such mode in delivering a self-management program for patients with both cardiac condition and diabetes. Furthermore, it will provide valuable information of refinement of the intervention program.

**Trial Registration Number:**

ACTRN12611000086965

## Background

Cardiac disease and diabetes are the most prevalent chronic diseases in the Australian population. The number of people with diabetes is estimated to increase from 171 million in 2000 to 366 million by 2030 [1,2]. The prevalence of diabetes for Australians aged 25 or over in 1999-2000 was 7.4% (more than 1 in 14 people) and the average age of people with diabetes is getting younger [3]. In 2000-2003, it is reported that 6.6% of Australians aged 18 years and over had diabetes [3]. Australia's Health 2008 confirmed people diagnosed with type 2 diabetes are more vulnerable to cardiovascular complications, and are at greater risk of further cardiac events, leading to poor health-related quality of life [3]. People with diabetes were reported to rate their health three times lower than those without diabetes [3,4]. The direct costs of treating type 2 diabetes and its related complications is estimated to reach between $213-396 billion a year globally by 2025, which is as high as 40 percent of some countries' health care budgets [5-9]. Therefore, reducing the risk of diabetes and its associated cardiac complications is vitally important not only from a personal, but also an economic perspective.

Two previous studies undertaken by the first two authors provided the basis for developing the initial Cardiac-Diabetes Self-Management Program (CDSMP). An exploratory study of the demographic characteristics and incidence of critical cardiac admission for patients with type 2 diabetes over a four-year period indicated 15% of all Coronary Care Unit (CCU) patients had a previous diagnosis of type 2 diabetes, and 22% of these patients were readmitted to hospital within 28 days compared to only 6% of CCU patients without diabetes [10]. A second study using a qualitative interpretative approach to gain in-depth understanding of the needs and experiences of this at-risk group of patients, found four main themes of confidence in self, confidence in health professionals, feelings of hopelessness and fatigue were related to managing their conditions following discharge [11]. These findings highlighted the complexity for patients with these two conditions in undertaking self-management. Both studies were invaluable in the development of a new self-management program, the CDSMP, which was based on self-efficacy construct [12].

The self-efficacy construct is based on the effect of a person's belief in their abilities to successfully follow through with planned actions and accomplish goals. Furthermore, a person with high self-efficacy will tend to set higher personal goals than someone with low self-efficacy, who will have very little faith in his/ her ability to achieve in the area in question [13].

Current literature supports the success of self-management programs based on improving self-efficacy levels in modifying lifestyles for different patient groups with chronic diseases such as type 2 diabetes [14,15] and cardiac disease [16]. Patients with type 2 diabetes who are admitted to CCU following a critical cardiac event are suddenly confronted with a number of life style rules and advice in order to deal with their illnesses. Often these rules and recommendations are presented separately for each disease and not tailored to the needs of patients with both diabetes and a cardiac condition. Furthermore, such guidance is given by professionals who might not have experienced any of the challenges these patients now face.

Having peer support during this initial period is very valuable. A recent World Health Organisation (WHO) consultation on peer support programs indicated these were promising and presented recommendations for developing and evaluating such programs [17]. A peer support person is someone who has experienced similar conditions, been successful in managing their conditions and is able to provide relevant and meaningful information [18].

Although the benefits of peer support for improving chronic illness self-management, including diabetes, to achieve better health outcomes has been demonstrated [18-22] there is limited evidence that self-management programs provided by or incorporating peers have improved psychological, or reduced health care utilization [22,23]. Furthermore, no research has yet been undertaken to evaluate the effectiveness of peer support for patients with both type 2 diabetes and cardiac conditions, nor has it been evaluated in different contexts (hospital and community).

## Aim

To develop and pilot test a Cardiac-Diabetes Self-Management Program (CDSMP) utilising peer support for improving self-management for patients with both diabetes and cardiac conditions.

### Objectives

• To further develop a CDSMP for patients with type 2 diabetes following a critical cardiac event, including the development of a DVD incorporating stories from a range of case studies, and use of former patients (peers) to provide support in adopting self-management strategies.

• To evaluate the feasibility and applicability of the DVD and peer supported CDSMP.

### Hypotheses

• Anticipated outcomes for the intervention group when compared to the control group receiving usual care will include:

• Higher self-management practice scores;

• Higher self-efficacy levels;

• Higher knowledge scores.

## Methods/Design

The pilot study using randomised controlled trial design (parallel-group trial) within the participants' usual health care environment will be conducted in three distinct but overlapping phases: (1) development of the training package including the CDSMP materials and Digital Video Disk (DVD), (2) peer selection and training, and (3) intervention phase. These will each be described below.

### Phase 1: Development of the CDSMP

The intervention utilised in this project will be based on the Cardiac Diabetes Self Management Program developed in earlier research by the first two authors [12]. It will be modified according to feedback from its initial trial, which included suggestions for adding a multimedia component; this will take the form of a DVD. The DVD will be provided to participants in the intervention group to assist them to apply strategies from the self-efficacy model; it will take the form of narrative "real-life" stories of people managing both diabetes and cardiac conditions. A further addition to the program will be the use of peers to provide follow-up support to participants.

The CDSMP program consists of three face-to-face sessions and telephone and text-messaging follow-up. The face-to-face sessions will be provided by a trained Research Nurse, and be conducted while the patients are in CCU and continue as the patient makes the transition home after discharge. The participants will be telephoned 1 week after discharge home and 2 text-messages will be sent a week after telephone follow up. These will be conducted by the trained 'peers'.

#### Reference Group

A reference group will be formed for the purposes of informing and reviewing program materials. The panel will consist of experts in the field (e.g. cardiologists, diabetes educators, cardiac rehabilitation coordinators) plus the researchers. After agreeing to participate, panellists will attend face-to-face meetings to establish the roles and responsibilities of the panel, such as reviewing and commenting on program development, including DVD production, and to agree on acceptable methods of communication. The panel will provide an important source of guidance in the production of all educational materials, in particular the DVD.

#### DVD Production

"Actors" in the DVD will be actual people dealing with diabetes and cardiac conditions and discussions will be held between these actors, the production team and Chief Investigator to ensure understanding of individual roles, as well as the DVD as a whole. Prior to production, reference group members will be given the opportunity to review and comment on the DVD script. The resulting DVD will be presented to the reference group for final approval before its use within the CDSMP.

### Phase 2: Peer Selection & Training

Selection and training of peer supporters is a crucial component to this project; a peer supporter is someone who has similar conditions, and can use their own experience to provide information to others. The peer support person can be very useful to others who are trying to work out the best ways of managing their health [18]. Potential peers, who are considered competent in the management of their conditions will be identified by the cardiologist and subsequently trained by the researchers. To facilitate the training process, a detailed peer training manual will be developed, also utilising self-efficacy principles.

### Phase 3: Intervention

Control group (group 1): Participants will receive usual care, which consists of education provided in the CCU and referral as necessary to the diabetes educator.

Intervention group (group 2): Participants will receive usual care and a combination of CDSMP sessions in the CCU, plus peer telephone and text follow-ups.

The CDSMP is designed to be administered on a one-to-one basis and to commence in CCU by staff members, then be followed up by trained peers via a telephone call one week after discharge and text message reminders from peers in weeks three and four. If a patient is transferred to a ward before completing the program, the hospital-based section of the CDSMP will be continued on the ward by the trained research nurse.

As part of the program, the DVD based on incorporating stories from a range of case studies is developed and will be provided to participants in the intervention group to assist them with the application of strategies from the Self-Efficacy Model.

#### Settings and locations

The study will be conducted in the Coronary Care Units (CCU) at Mater Health Services, and followed up by telephone and text messages at participants' homes in Brisbane, Australia.

## Participants

### Sample size

A total of 30 patients for 2 groups will be required. This sample size of 10-15 per group is suggested by Hertzog (2008) [19]. This number is calculated with an alpha of 0.05 and power of 0.9, thus 10 patients per group will be required to detect any statistical differences. Allowing 20% attrition rate, 13 patients per group will be required. Thus with an alpha of 0.05 and power of 0.9, 8 patients per group was required to detect any statistical differences.

#### Inclusion criteria

Participants who will be included:

• are males and females aged over 18 years who have given their consent to participate in the study

• have had type 2 diabetes for at least one year and have been admitted to the CCU with a critical cardiac event in the study hospitals

• can read and converse in English

• are physically stabilised

• have a mobile phone

#### Exclusion criteria

• Potential participants will be excluded if they:

• are unable to read and speak English

• are transferred to another hospital.

• are on respiratory ventilation

• are unconscious

Minority groups or people from non-English speaking backgrounds (NESB) will be included unless they cannot read and speak English. English is needed, as the materials are in English. Programs for non-English speaking patients will be developed in future projects.

### Randomisation

Simple randomisation using a randomisation table (computerised sequence generation) will be used. Allocation will be concealed by using sealed, numbered, opaque envelopes to both control and intervention groups.

## Data Collection

Primary and secondary outcomes: Primary outcomes include self-efficacy, self-care behaviour and knowledge. Secondary outcome is readmission rate within 28 days.

Baseline (Time 1): When patients' physical condition is stable they will be invited by a Research Assistant (RA) to participate in the study. Following explanation of the study, demographic and baseline data will be collected for those giving informed consent.

One month follow-up (Time 2): A questionnaire will be posted to participants at their discharge location, after which the RA will telephone the participant and collect data over the phone. Posting the questionnaire first will facilitate data collection in the telephone interview. This method has been used previously by a research team member [24], resulting in a high response rate (84%).

### Measures

Data will be collected on primary outcomes of this study: self-management practice, self-efficacy, knowledge and secondary outcome: readmission rates.

#### Self-management practice

Data will be collected at Time 1 and Time 2, and will be measured using the Summary of Diabetes Self-Care Activities (SDSCA) [25]. The SDSCA comprises 11 items that measure patients' adherence to self-management activities such as diet, exercise, blood glucose testing, foot care, and smoking status. The respondents will be asked to rate each item using 0-7 scale, with 7 indicating most adherence to each aspect, and 0 for non-compliance. Permission to use this tool has been granted by the authors.

#### Self-efficacy

Data will be collected at Time 1 and Time 2, and will be measured using the Diabetes Management Self-Efficacy Scale (DMSES) (Australian-English version) [26]. The DMSES comprises 20 items that measure patients' confidence levels (self-efficacy) in managing their diabetes. The respondents will be asked to rate each item using 0-10 scale, with 10 indicating the highest confidence level, and 0 the lowest. This scale is designed to measure the self-efficacy levels of patients with type 2 diabetes. A high internal consistency was demonstrated by the Cronbach alpha coefficient of 0.9. Face validity was established through consultation with diabetes educators and patients with type 2 diabetes, and convergent validity was demonstrated by the significant correlations between DMSES and the General Self-Efficacy Scale (GSE). Permission to use this tool has been granted.

#### Knowledge

Data will be collected at Time 1 and Time 2, and will be measured using Diabetes Knowledge Questions (DKQ) [27]. The DKQ measures patients' knowledge of diabetes and cardiac conditions. Each item with a correct answer scores one point; score ranges are therefore 0 to 8. Cronbach alpha for DKQ is 0.7, indicating good internal consistency [27]. In addition, this scale is brief and unlikely to cause a 'response burden'; particularly as patients who have diabetes and have had a cardiac condition may become tired answering questions. Permission to use this tool has been granted.

#### Readmission rate

Readmission rate will be collected at Time 2, and will be ascertained from hospital records.

### Procedure

**Figure 1:**
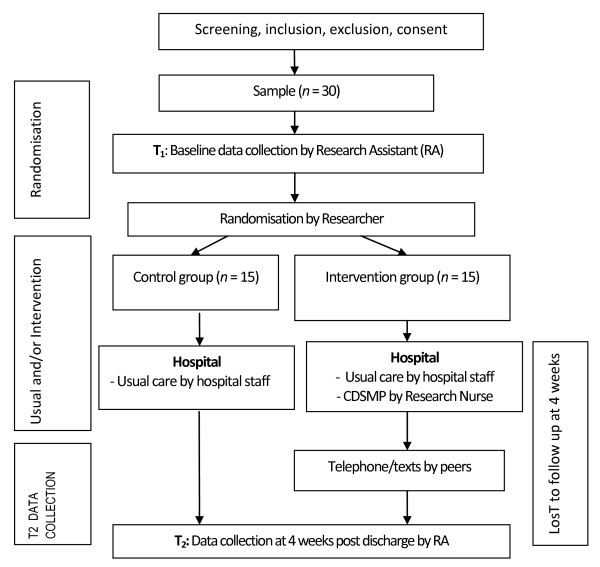
**Overview of Data Collection**.

The nominated RA will liaise with the Nurse Unit Manager (NUM) of the CCU, to obtain a list of eligible participants from the CCU Admission Book. The RA will then approach the potential participants for their informed consent and to collect baseline data. After baseline data is collected, a computer-generated list of random numbers produced by the researcher will then be used to randomly assign participants to either the control group or the intervention group (See Figure 1: Overview of data collection). Patients are blinded to receive either usual care or usual care and CDSMP, however the RA who assisting with data analysis is not blinded.

Control group (group 1): Participants will receive usual care, which consists of education provided in the CCU and referral as necessary to the diabetes educator.

Intervention group (group 2): Participants will receive usual care and a combination of CDSMP sessions in the CCU, plus peer telephone and text follow-ups. The CDSMP was designed to be administered on a one-to-one basis and to commence in CCU by staff members, then be followed up by trained peers via a telephone call one week after discharge and text message reminders from peers in weeks three and four. If a patient is transferred to a ward before completing the program, the hospital-based section of the CDSMP will be continued on the ward by the trained research nurse.

As part of the program, the DVD based on incorporating stories from a range of case studies is developed and will be provided to participants in the intervention group to assist them with the application of strategies from the Self-Efficacy Model.

## Data Analysis

Prior to undertaking statistical tests, the homogeneity of the sample will be examined. Due to small samples size; non-parametric statistical tests will be used to determine the relationships and differences between disease and demographic variables: Chi-square for categorical variables and Mann-Whitney U test for continuous variables. The Wilcoxon Signed Rank test will be used to determine whether any changes in outcome variables occurred within and between groups over time. Mann-Whitney U test will be used to confirm whether there is an interaction between time and group. All analyses will be conducted using SPSS version 18 with a pre-defined level of statistical significance at p < 0.05.

## Ethical Considerations

Ethical approvals have been granted by the Mater Health Services Human Research Ethics Committee and Queensland University of Technology Human Research Ethics Committee.

Informed consent: All participants will be volunteers who will provide written consent, and will be free to withdraw at any time. Confidentiality: No identifying information will be kept with the data, which will be analysed and stored on a secure network drive at the university, protected by password and used only for the purposes of this study. Only the researchers involved in this study will have access to the data. While no information about the project will be published in any form that will allow any individual to be recognised; non-identifiable results will be reported within the hospital and in the literature.

This study protocol is now registered with the Australia and New Zealand Clinical Trials Registry (Trial Number: ACTRN12611000086965).

## Discussion

This protocol has provided information on planning and pilot testing the feasibility of a peer-support based cardiac-diabetes self-management program. This project aims to compare and evaluate transitional care interventions in order to reduce readmission rates and emergency health service visits by patients with diabetes and a cardiac event, and to improve their health, and psychosocial well-being. Additionally, this project seeks to improve the uptake of appropriate self-management in people with both diabetes and a cardiac condition, and encourage maintenance of independence. The findings of this pilot study will provide baseline knowledge for undertaking a larger study to test the effectiveness of a self-management program, which incorporates text messaging and telephone follow-up by a peer support person for patients with both diabetes and a cardiac condition.

## Limitations

The results of this pilot study will provide valuable information of the feasibility and acceptability of the peer-support based cardiac-diabetes self-management program, and direction of power analysis for required sample size for a larger study. However, staff variations and trend of patients within participating study sites may vary for a future full study. For example, patients' self-care behaviour and self-efficacy levels may not be consistent with pilot study. The research nurse trained for this study is specialised in coronary care, which could possibly lead to contamination associated with delivering the program.

## Conclusions

The proposed peer-support based self-management program uses self-efficacy construct of Bandura's Social Cognitive Theory which is flexible and could potentially be adapted in other chronic diseases. This intervention, incorporating peers and telephone and text-messaging approach will provide important information for future research and care planning.

## Competing interests

The authors declare that they have no competing interests.

## Authors' contributions

CJW, the principal investigator of the study, conducted the data analysis with oversight of co-authors: AMC, MC, LMSB, and KK, drafted the initial manuscript. All co-authors reviewed and approved the final draft. KK participated in preparation of selecting and training peers, and critically reviewed, edited and approved the final draft.

## Pre-publication history

The pre-publication history for this paper can be accessed here:

http://www.biomedcentral.com/1472-6963/11/74/prepub
